# The correlations of serum uric acid with lean mass, fat mass and grip strength in adolescents aged 12-19 years

**DOI:** 10.3389/fendo.2024.1507643

**Published:** 2025-02-13

**Authors:** Feng Xu, Jianjun Shen, Zhongxin Zhu

**Affiliations:** ^1^ Department of Clinical Laboratory, The First People’s Hospital of Xiaoshan District, Xiaoshan Affiliated Hospital of Wenzhou Medical University, Hangzhou, Zhejiang, China; ^2^ Department of General Practice, Community Health Service Center of Guali, Hangzhou, Zhejiang, China; ^3^ Department of Osteoporosis Care and Control, The First People’s Hospital of Xiaoshan District, Xiaoshan Affiliated Hospital of Wenzhou Medical University, Hangzhou, Zhejiang, China

**Keywords:** uric acid, body composition, muscle strength, adolescent, NHANES

## Abstract

**Background:**

Serum uric acid (sUA) has emerged as an intriguing modulator of body composition and physical function, yet its complex associations with musculoskeletal parameters during the critical period of adolescence remain incompletely characterized. To address this knowledge gap, we sought to elucidate the relationships between sUA and key indicators of body composition and musculoskeletal health in adolescents aged 12-19 years, specifically examining appendicular lean mass index (ALMI), appendicular fat mass index (AFMI), and combined grip strength.

**Methods:**

In this cross-sectional study, we analyzed data from 2,003 adolescents participating in the National Health and Nutrition Examination Survey (NHANES) 2011-2014. We examined the relationships between sUA and ALMI, AFMI, and combined grip strength using multivariate linear regression models. Subgroup analyses were conducted to explore effect modifications by age, sex, and race/ethnicity.

**Results:**

Higher sUA levels were positively associated with ALMI and grip strength, and inversely associated with AFMI after adjusting for potential confounders. These associations exhibited distinct patterns across age, sex, and race subgroups, with the most pronounced effects observed among boys aged 12-15 years and in non-Hispanic White and Black populations.

**Conclusions:**

Our findings demonstrated significant associations between sUA levels and various parameters of musculoskeletal health and body composition, suggesting that sUA may serve as a potential biomarker for monitoring physical development and maturation during adolescence.

## Introduction

Adolescence marks a critical window of physical development, during which dramatic changes in body composition and muscular capabilities occur (1). During this period, various physiological factors orchestrate musculoskeletal development, with serum uric acid (sUA) emerging as an intriguing yet understudied mediator (2). While traditionally linked to metabolic disorders, mounting evidence suggests that uric acid may play broader roles in human physiology, particularly in body composition regulation and muscle function (3, 4).

As the predominant antioxidant in human serum, uric acid accounts for approximately 60% of free radical scavenging activity (5). This property could theoretically protect muscle tissue and influence its development (6). Moreover, sUA interacts with key metabolic pathways affecting insulin sensitivity and glucose homeostasis, potentially impacting both muscle and fat tissue dynamics (7, 8). However, the relationship between sUA and body composition parameters in adolescents remains unclear - a significant concern given rising rates of youth obesity and hyperuricemia (9, 10).

Evidence linking sUA to muscle mass and strength has been inconsistent. Some studies report positive associations (11–13), while others show no protective effects (14, 15). These contradictions underscore the need to better understand sUA’s role during adolescent development. Despite extensive research on traditional factors like exercise and nutrition in adolescent musculoskeletal health, sUA’s influence during this critical period remains largely unexplored.

To address this knowledge gap, we sought to elucidate the relationships between sUA and key indicators of musculoskeletal health and body composition in adolescents aged 12-19 years. Leveraging a large sample from the National Health and Nutrition Examination Survey (NHANES), we conducted a comprehensive analysis of the correlations of sUA with appendicular lean mass index (ALMI), appendicular fat mass index (AFMI), and grip strength.

## Methods

### Study design and population

We conducted a cross-sectional analysis of data from NHANES 2011-2014 cycles. NHANES, administered biennially by the National Center for Health Statistics (NCHS), provides comprehensive health and nutritional data on the U.S. population. The study protocol was approved by the NCHS Research Ethics Review Board, and all participants or their legal guardians provided written informed consent.

Our initial sample comprised 2,705 adolescents aged 12-19 years. After excluding individuals with incomplete sUA data (n=393), ALMI and AFMI measurements (n=265), and combined grip strength assessments (n=44), the final analytical sample consisted of 2,003 subjects.

### Exposure and outcome variables

The exposure variable was sUA, which was measured using the Beckman Coulter UniCel^®^ DxC800 system during the 2011-2014 survey cycles. The outcome variables included ALMI, AFMI, and combined grip strength. ALMI and AFMI were derived from dual-energy X-ray absorptiometry (DXA) scans performed using a Hologic QDR-4500A fan-beam densitometer (Hologic, Inc., Bedford, MA). These indices were calculated as appendicular lean/fat mass [kg] divided by height squared [m²]. Grip strength was assessed using a Takei Digital gripper force gauge (model T.K.K.5401), following a standardized protocol. Our analysis utilized the combined grip strength, representing the sum of the largest reading from each hand.

### Confounding variables

We identified potential confounders based on clinical insights and prior research (12, 16, 17): age (12-15 and 16-19 years), sex, race/ethnicity, family income-to-poverty ratio, moderate activity, body mass index (BMI), nutritional parameters (dietary protein, vitamin D, calcium intake), and serum markers (blood urea nitrogen, total protein, serum calcium). Moderate activity was assessed through self-reported moderate-intensity activities, and dietary intakes were obtained from two 24-hour dietary recall interviews.

### Statistical analyses

We stratified subjects by age and sex groups, presenting baseline characteristics as means ± standard deviations for continuous variables and percentages for categorical variables. Inter-group differences were assessed using appropriate statistical tests: χ² for categorical variables, one-way ANOVA for normally distributed continuous variables, and Kruskal-Wallis H tests for skewed distributions.

To evaluate associations between sUA and ALMI, AFMI, and grip strength, we employed multivariate linear regression models. Following STROBE statement recommendations (18), we constructed three models: unadjusted, partially adjusted (for age, sex, and race), and fully adjusted (for all screened covariates). Subgroup analyses stratified by age, sex, and race/ethnicity were further performed using stratified linear regression models to explore potential effect modifications, with interaction terms tested using likelihood ratio tests.

To explore and confirm potential non-linear associations, we employed smooth curve fitting techniques and generalized additive models, allowing for the detection of nuanced relationships that might not be captured by linear models alone.

All statistical analyses were conducted using R software (version 3.4.3) and EmpowerStats (X&Y Solutions, Inc., Boston, MA). Statistical significance was set at P < 0.05 (two-sided).

## Results

### Demographic and clinical characteristics

The characteristics of 2,003 adolescents aged 12-19 years stratified by age and sex are presented in **Table 1**. Significant inter-group variations were observed in race/ethnicity distribution and engagement in moderate activities. Older adolescents exhibited higher BMI, while boys demonstrated greater dietary intakes compared to girls. Serum biomarkers showed significant variations among groups. Notably, boys displayed higher ALMI and combined grip strength (both P < 0.001), whereas girls had higher AFMI (P < 0.001).

**Table 1:** Characteristics of study population based on age and sex group.

Age/sex group	12-15y boys (n=537)	12-15y girls (n=490)	16-19y boys (n=500)	16-19y girls (n=476)	P value
Race/Ethnicity (%)					<0.001
Non-Hispanic White	25.0	23.9	27.8	22.5	
Non-Hispanic Black	27.2	25.1	25.0	26.5	
Mexican American	22.0	22.2	20.0	22.5	
Other race/ethnicity	25.9	28.8	27.2	28.6	
Moderate activities (%)					<0.001
Yes	53.1	52.9	47.8	42.9	
No	44.3	45.9	52.0	57.1	
Unrecorded	2.6	1.2	0.2	0.0	
Ratio of family income to poverty	2.1 ± 1.6	2.1 ± 1.5	2.0 ± 1.6	1.8 ± 1.5	0.003
Body mass index (kg/m^2^)	22.6 ± 5.7	23.6 ± 5.9	25.4 ± 6.0	25.3 ± 6.7	<0.001
Dietary protein intake (g/d)	81.9 ± 33.5	60.9 ± 24.9	94.5 ± 47.7	65.6 ± 24.7	<0.001
Dietary vitamin D intake (μg/d)	8.1 ± 7.8	6.9 ± 23.8	7.9 ± 11.3	6.2 ± 8.2	<0.001
Dietary calcium intake (mg/d)	1117.9 ± 523.9	844.6 ± 439.9	1176.4 ± 623.3	867.6 ± 439.5	<0.001
Blood urea nitrogen (mmol/L)	3.8 ± 1.1	3.4 ± 1.2	4.1 ± 1.2	3.6 ± 1.0	<0.001
Total protein (g/L)	72.0 ± 4.2	72.0 ± 4.2	73.2 ± 4.3	72.7 ± 4.3	<0.001
Serum calcium (mmol/L)	2.42 ± 0.07	2.40 ± 0.07	2.42 ± 0.08	2.38 ± 0.07	<0.001
Serum uric acid (umol/L)	313.8 ± 67.2	264.3 ± 57.1	350.8 ± 66.5	261.9 ± 57.3	<0.001
Appendicular lean mass index (kg/m^2^)	7.4 ± 1.4	6.4 ± 1.3	8.5 ± 1.5	6.6 ± 1.4	<0.001
Appendicular fat mass index (kg/m^2^)	3.4 ± 1.9	4.6 ± 1.9	3.3 ± 1.8	4.9 ± 2.0	<0.001
Combined grip strength (kg)	62.8 ± 17.1	51.3 ± 10.1	85.3 ± 14.7	56.8 ± 9.7	<0.001

### Correlations of sUA with ALMI, AFMI, and combined grip strength

Multivariate regression analyses revealed complex relationships between sUA and musculoskeletal health and body composition parameters (**Table 2**). SUA demonstrated a robust positive association with ALMI across all models (β = 0.002, 95% CI: 0.001-0.002, P < 0.001 in fully adjusted model). For AFMI, initial positive associations in minimally adjusted models shifted to a negative association in the fully adjusted model (β = -0.002, 95% CI: -0.003 to -0.002, P < 0.001). Combined grip strength exhibited a consistent positive association with sUA (β = 0.035, 95% CI: 0.025-0.045, P < 0.001 in fully adjusted model). Comparing the highest (Q4) to lowest (Q1) quartile of sUA revealed significant increases in ALMI and combined grip strength, with a concomitant decrease in AFMI in the fully adjusted model (all P for trend < 0.001).

**Table 2:** Association of serum uric acid (umol/L) with ALMI (kg/m^2^), AFMI (kg/m^2^), and combined grip strength (kg).

	Model 1 β (95% CI)	Model 2 β (95% CI)	Model 3 β (95% CI)
ALMI	0.011 (0.011, 0.012) ^***^	0.009 (0.008, 0.010) ^***^	0.002 (0.001, 0.002) ^***^
Serum uric acid Q4			
Q1	Reference	Reference	Reference
Q2	0.554 (0.376, 0.732)	0.444 (0.287, 0.601)	0.123 (0.039, 0.207)
Q3	1.187 (1.009, 1.366)	0.872 (0.707, 1.037)	0.220 (0.130, 0.310)
Q4	2.217 (2.040, 2.394)	1.694 (1.519, 1.868)	0.359 (0.257, 0.461)
P for trend	<0.001	<0.001	<0.001
AFMI	0.003 (0.002, 0.004) ^***^	0.010 (0.009, 0.012) ^***^	-0.002 (-0.003, -0.002) ^***^
Serum uric acid Q4			
Q1	Reference	Reference	Reference
Q2	-0.025 (-0.282, 0.233)	0.350 (0.116, 0.583)	-0.161 (-0.251, -0.071)
Q3	-0.066 (-0.324, 0.193)	0.836 (0.591, 1.081)	-0.272 (-0.369, -0.176)
Q4	0.512 (0.255, 0.768)	1.828 (1.568, 2.088)	-0.420 (-0.529, -0.311)
P for trend	<0.001	<0.001	<0.001
Combined grip strength	0.119 (0.109, 0.129) ^***^	0.054 (0.045, 0.062) ^***^	0.035 (0.025, 0.045) ^***^
Serum uric acid Q4			
Q1	Reference	Reference	Reference
Q2	5.637 (3.544, 7.730)	2.817 (1.194, 4.441)	1.902 (0.322, 3.482)
Q3	13.566 (11.469, 15.662)	5.981 (4.276, 7.686)	4.223 (2.527, 5.919)
Q4	22.951 (20.872, 25.030)	10.557 (8.749, 12.364)	7.014 (5.101, 8.927)
P for trend	<0.001	<0.001	<0.001

Model 1: no covariates were adjusted.

Model 2: age, sex and race were adjusted.

Model 3: age, sex, race, ratio of family income to poverty, moderate activities, body mass index, dietary protein, vitamin D and calcium intake, blood urea nitrogen, total protein, and serum calcium were adjusted.

^*^P <0.05, ^**^P <0.01, ^***^P <0.001.

ALMI, appendicular lean mass index; AFMI, appendicular fat mass index.

### Non-linear relationships and subgroup analyses

**Figure 1:**
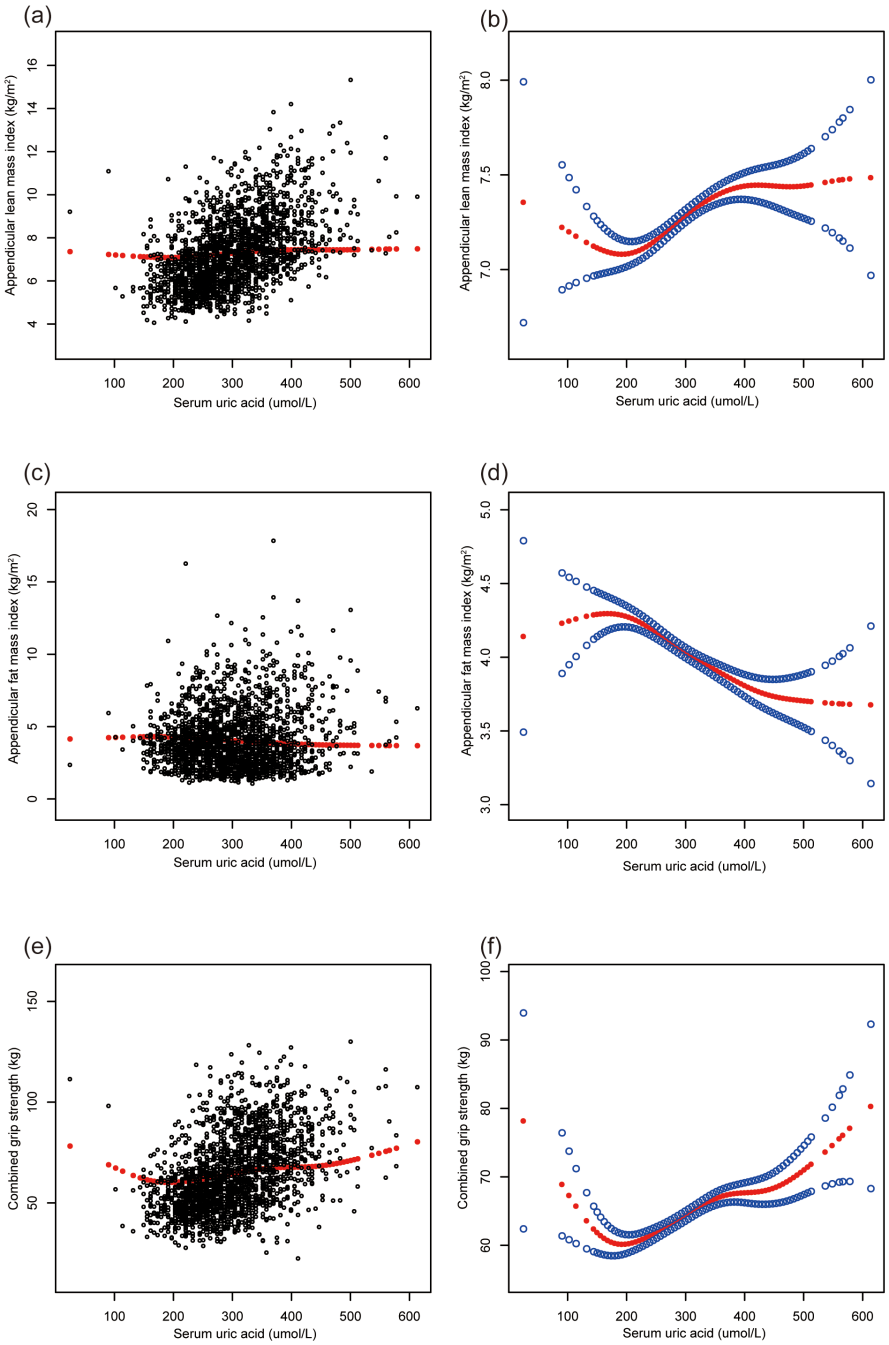
The association of serum uric acid with ALMI, AFMI and combined grip strength. **(A, B)** ALMI; **(C, D)** AFMI; **(E, F)** combined grip strength. ALMI, appendicular lean mass index; AFMI, appendicular fat mass index. Age, sex, race, ratio of family income to poverty, moderate activities, body mass index, dietary protein, vitamin D and calcium intake, blood urea nitrogen, total protein, and serum calcium were adjusted.

**Figure 1** illustrates the relationships between sUA and musculoskeletal health and body composition parameters in adolescents. Positive, non-linear relationships were observed between sUA, ALMI, and combined grip strength, while a negative association was evident with AFMI.

Subgroup analyses (**Table 3**) uncovered heterogeneous associations across age, sex, and race/ethnicity groups. ALMI showed positive associations in 12-15y boys, non-Hispanic White, and non-Hispanic Black groups. AFMI demonstrated negative associations in 12-15y boys, 16-19y girls, and all race/ethnicity groups. Combined grip strength showed positive associations in 12-15y boys, non-Hispanic White, and non-Hispanic Black groups.

**Table 3:** Subgroup analysis of the associations between serum uric acid (umol/L), ALMI (kg/m^2^), AFMI (kg/m^2^) and combined grip strength (kg).

	No. of participants	β (95% CI)	P for interaction
ALMI			
Age, sex			<0.001
12−15y boy	537	0.003 (0.003, 0.004)	
12−15y girl	490	0.000 (−0.001, 0.002)	
16−19y boy	500	−0.001 (−0.002, 0.000)	
16−19y girl	476	0.001 (−0.000, 0.002)	
Race/Ethnicity			0.015
Non−Hispanic White	497	0.002 (0.001, 0.003)	
Non−Hispanic Black	520	0.002 (0.001, 0.003)	
Mexican American	434	0.000 (−0.001, 0.002)	
Other race/ethnicity	552	0.001 (−0.000, 0.002)	
AFMI			
Age, sex			<0.001
12−15y boy	537	−0.004 (−0.005, −0.003)	
12−15y girl	490	−0.001 (−0.002, 0.001)	
16−19y boy	500	0.000 (−0.001, 0.001)	
16−19y girl	476	−0.002 (−0.003, −0.001)	
Race/Ethnicity			0.213
Non−Hispanic White	497	−0.003 (−0.004, −0.001)	
Non−Hispanic Black	520	−0.003 (−0.004, −0.002)	
Mexican American	434	−0.001 (−0.002, −0.000)	
Other race/ethnicity	552	−0.002 (−0.003, −0.001)	
Combined grip strength			
Age, sex			<0.001
12−15y boy	537	0.081 (0.064, 0.098)	
12−15y girl	490	0.000 (−0.022, 0.022)	
16−19y boy	500	0.006 (−0.013, 0.024)	
16−19y girl	476	0.001 (−0.020, 0.023)	
Race/Ethnicity			0.002
Non−Hispanic White	497	0.049 (0.031, 0.068)	
Non−Hispanic Black	520	0.057 (0.037, 0.076)	
Mexican American	434	0.013 (−0.007, 0.033)	
Other race/ethnicity	552	0.020 (0.003, 0.038)	

Age, sex, race, ratio of family income to poverty, moderate activities, body mass index, dietary protein, vitamin D and calcium intake, blood urea nitrogen, total protein, and serum calcium were adjusted. In the subgroup analysis, the model is not adjusted for the stratification variable itself.

ALMI, appendicular lean mass index; AFMI, appendicular fat mass index.

**Figure 2:**
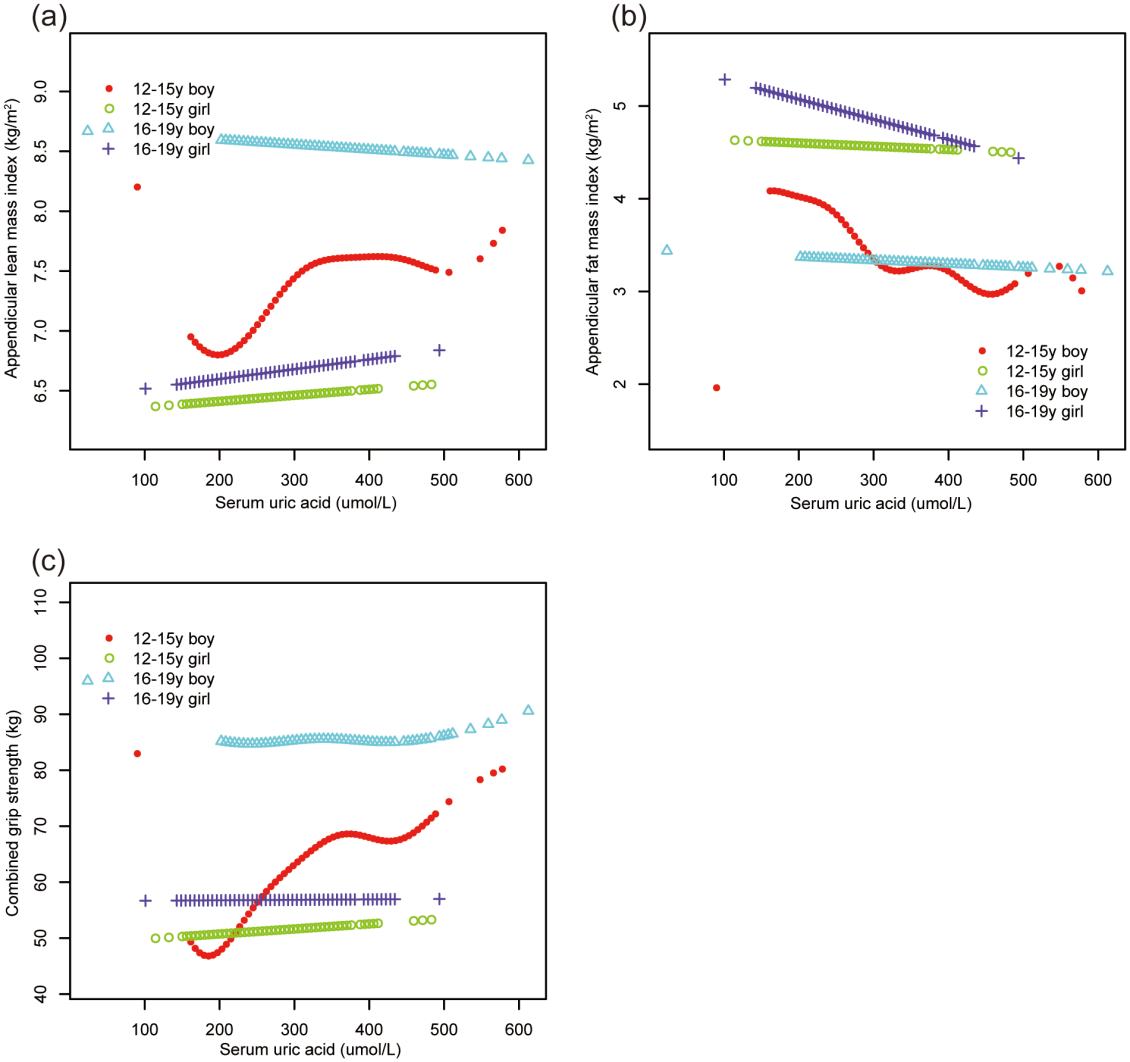
The association of serum uric acid with ALMI, AFMI and combined grip strength, stratified by age/sex. **(A)** ALMI; **(B)** AFMI; **(C)** combined grip strength. ALMI, appendicular lean mass index; AFMI, appendicular fat mass index. Race, ratio of family income to poverty, moderate activities, body mass index, vitamin D intake and calcium intake were adjusted.

**Figure 3:**
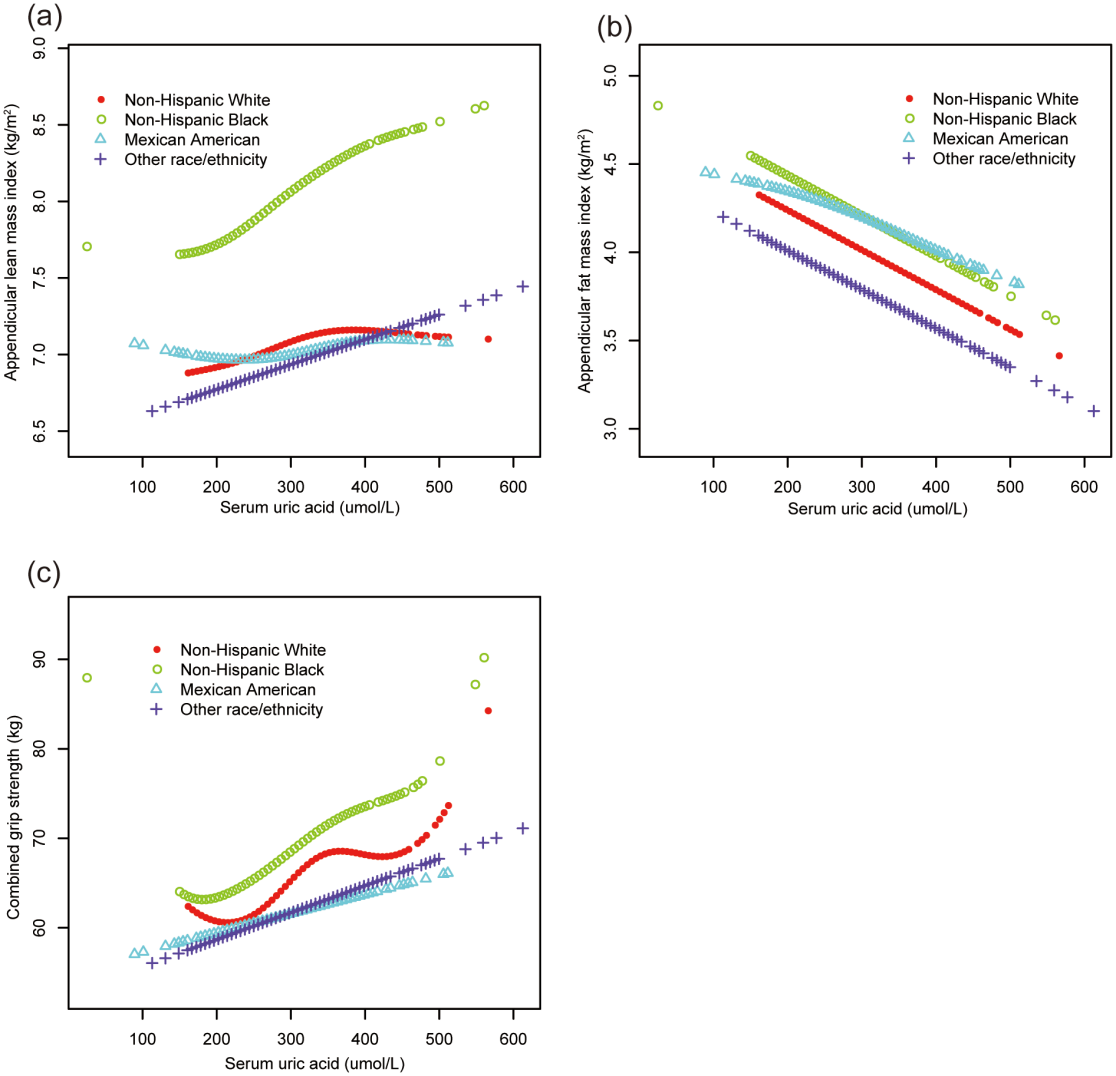
The association of serum uric acid with ALMI, AFMI and combined grip strength, stratified by race. **(A)** ALMI; **(B)** AFMI; **(C)** combined grip strength. ALMI, appendicular lean mass index; AFMI, appendicular fat mass index. Age, sex, ratio of family income to poverty, moderate activities, body mass index, vitamin D intake and calcium intake were adjusted.

Smooth curve fittings (**Figures 2**, **3**) further corroborated these stratified associations between sUA and body composition parameters, elucidating complex, non-linear relationships.

## Discussion

Our analysis of 2,003 adolescents aged 12-19 years revealed complex associations between sUA and key indicators of musculoskeletal health and body composition. We found significant correlations between sUA and ALMI, AFMI, and grip strength, with distinct patterns across demographic subgroups.

Previous studies have reported conflicting relationships between sUA and muscle parameters. While positive correlations between sUA and muscle mass were observed in kidney transplant recipients (19), elevated sUA predicted reduced muscle mass in men with type 2 diabetes (20). Similarly divergent findings emerged in Chinese populations, with both positive (13) and negative (21) associations reported, suggesting demographic and health-specific modulation of these relationships.

The sUA-muscle strength relationship shows comparable complexity. Studies have reported positive correlations in elderly Japanese women (22), inverted J-shaped associations in Chinese adults (23) and Japanese men (24), and inverse correlations in Korean women (15). Health status further influences this relationship, as evidenced by strength improvements despite increased sUA in diabetic patients undergoing resistance training (25).

Notably, our analysis revealed a negative association between sUA and AFMI in fully adjusted models, contrasting with previous adult studies linking elevated sUA to increased adiposity (26–28). This finding suggests age-specific dynamics in uric acid metabolism and body composition regulation during adolescence.

Subgroup analyses unveiled marked demographic variations. The positive associations between sUA and both ALMI and grip strength were most pronounced in boys aged 12-15 years and non-Hispanic White and Black participants. The varying patterns across racial groups suggest potential genetic and environmental modifiers, including differences in dietary patterns, physical activity levels, and genetic variants affecting uric acid metabolism. Sex emerged as a crucial modifier, consistent with recent findings showing sex-specific associations between sUA and sarcopenia risk (29). This highlights the need for age, sex, and race specific reference ranges when considering sUA as a clinical marker.

The differential associations between sUA and musculoskeletal health and body composition parameters may be explained by several physiological mechanisms unique to adolescent development. The positive association between sUA and lean mass may be attributed to uric acid’s antioxidant properties (30), which could protect developing muscle tissue from oxidative stress during rapid growth. Additionally, sUA’s potential influence on protein synthesis regulation may be particularly relevant during adolescence, when muscle protein turnover is enhanced. Conversely, the inverse relationship between sUA and fat mass may reflect the intricate interplay between uric acid metabolism and adipose tissue function, potentially mediated through inflammatory pathways and adipokine signaling that are distinctively active during adolescent growth (31). Future research should focus on establishing causality through longitudinal studies and examining whether maintaining optimal sUA levels during adolescence could promote healthy musculoskeletal development.

This study analyzed data from 2,003 adolescents aged 12-19 years, enhancing the generalizability of our findings. Methodologically, DXA scans for body composition assessment and standardized grip strength measurements were employed in this study, ensuring reliable and accurate outcome measures. Despite these strengths, several limitations warrant consideration. Primarily, the cross-sectional design precludes the establishment of causal relationships between sUA and musculoskeletal health parameters. Additionally, despite adjusting for numerous confounders, the possibility of residual confounding persists; factors such as pubertal stage, unavailable in the NHANES dataset, could potentially modulate the observed associations. Third, our study focused exclusively on adolescents aged 12-19 years from the general population. While this provides valuable insights into the relationships of sUA with musculoskeletal health and body composition during this critical developmental period, our findings may not be generalizable to populations outside this age range or those with specific medical conditions. Lastly, the single time-point assessment of sUA and related parameters may inadequately capture their dynamic nature, particularly during the rapidly evolving period of adolescence, underscoring the need for longitudinal investigations.

## Conclusion

Our analysis demonstrated significant associations between sUA levels and various parameters of musculoskeletal health and body composition, suggesting sUA’s potential utility as a developmental biomarker. The differential associations observed across age, sex, and race groups indicated the need for demographic-specific reference ranges when considering sUA as a clinical indicator. Future research should focus on specific topics such as the influence of physical activity and dietary factors on sUA levels, as well as longitudinal studies to establish potential causal relationships and elucidate the temporal dynamics of these associations.

## Data Availability

The datasets presented in this study can be found in online repositories. The data of this study are publicly available on the NHANES website (https://www.cdc.gov/nchs/nhanes/index.htm).
